# Inequity in costs of seeking sexual and reproductive health services in India and Kenya

**DOI:** 10.1186/s12939-015-0216-5

**Published:** 2015-09-15

**Authors:** Hassan Haghparast-Bidgoli, Anni-Maria Pulkki-Brännström, Yves Lafort, Mags Beksinska, Letitia Rambally, Anuradha Roy, Sushena Reza-Paul, Wilkister Ombidi, Peter Gichangi, Jolene Skordis-Worrall

**Affiliations:** Institute for Global Health, University College London, London, UK; Epidemiology and Global Health, Umeå University, Umeå, Sweden; International Centre for Reproductive Health, Ghent University, Ghent, Belgium; MatCH (Maternal, Adolescent and Child Health), University of the Witwatersrand, Durban, South Africa; Ashodaya Samithi (Ashodaya), Mysore, India; International Centre for Reproductive Health Association (ICRHK), Mombasa, Kenya

**Keywords:** Sexual and reproductive health services, Inequity, Kakwani index, Catastrophic spending

## Abstract

**Objective:**

This study aims to assess inequity in expenditure on sexual and reproductive health (SRH) services in India and Kenya. In addition, this analysis aims to measure the extent to which payments are catastrophic and to explore coping mechanisms used to finance health spending.

**Methods:**

Data for this study were collected as a part of the situational analysis for the “Diagonal Interventions to Fast Forward Enhanced Reproductive Health” (DIFFER) project, a multi-country project with fieldwork sites in three African sites; Mombasa (Kenya), Durban (South Africa) and Tete (Mozambique), and Mysore in India. Information on access to SRH services, the direct costs of seeking care and a range of socio-economic variables were obtained through structured exit interviews with female SRH service users in Mysore (India) and Mombasa (Kenya) (*n* = 250). The costs of seeking care were analysed by household income quintile (as a measure of socio-economic status). The Kakwani index and quintile ratios are used as measures of inequitable spending. Catastrophic spending on SRH services was calculated using the threshold of 10 % of total household income.

**Results:**

The results showed that spending on SRH services was highly regressive in both sites, with lower income households spending a higher percentage of their income on seeking care, compared to households with a higher income. Spending on SRH as a percentage of household income ranged from 0.02 to 6.2 % and 0.03–7.5 % in India and Kenya, respectively. There was a statistically significant difference in the proportion of spending on SRH services across income quintiles in both settings. In India, the poorest households spent two times, and in Kenya ten times, more on seeking care than the least poor households. The most common coping mechanisms in India and Kenya were “receiving [money] from partner or household members” (69 %) and “using own savings or regular income” (44 %), respectively.

**Conclusion:**

Highly regressive spending on SRH services highlights the heavier burden borne by the poorest when seeking care in resource-constrained settings such as India and Kenya. The large proportion of service users, particularly in India, relying on money received from family members to finance care seeking suggests that access would be more difficult for those with weak social ties, small social networks or weak bargaining positions within the family - although this requires further study.

## Introduction

Out-of-pocket expenditures (OOPE) continue to be the main source of financing for health care in many low- and middle-income countries (LMICs), in particular countries in Asia and Africa [1–4]. In these countries OOPE represent more than 50 % of health care spending [1]. OOPE has been found to be a financial barrier to accessing essential care, to increase the risk of more severe or longer term ill-health, to place households at increased risk of poverty and to exacerbate inequity [1, 5].

Recent evidence from Africa and Asia shows that high OOPE discourages individuals from poor and disadvantaged groups from seeking health care [1, 2, 6]. Even relatively modest levels of absolute spending can be catastrophic for poorer households, forcing them to reduce spending on other essential items such as food or to rely on high-risk coping strategies (e.g., selling productive assets) [7–9]. Based on World Health Organization (WHO) estimates, every year approximately 44 million households worldwide (or more than 150 million individuals) face catastrophic health expenditure, and about 25 million households (or more than 100 million individuals) are pushed into poverty by the need to pay for health services [10].

The financial burden of sexual and reproductive health (SRH) care seeking in LMICs has not been widely explored in the literature. Few studies have measured OOPE or the equity impact of payments for SRH in LMICs. Among available studies, most have focused on maternal care [11–15]. The evidence from these studies suggests that costs of seeking SRH services could be significant and catastrophic, and affect more individuals from low socio-economic groups.

Poor sexual and reproductive health accounts for a large share of the global burden of disease, disproportionally affecting LMICs [16–19]. Addressing the financial barriers to SRH care access is also critical to improving progress towards Millennium Development Goal 5 *(*MDG 5: Reduce Maternal Mortality & Achieve Universal Access to Reproductive Health), MDG 6 (Combat HIV/AIDS) and to some extent MDG 3 (Promote gender equality and empower women) and MDG 4 (Reduce Child Mortality). However, addressing financial barriers to essential health care access will be challenging for many low- and middle-income countries, particularly those such as India and Kenya, which lack universal pre-payment mechanisms for health care, have high degrees of relative poverty (inequality) and high levels of absolute poverty [2, 7, 20, 21].

This study aims to explore three main characteristics of inequality in spending on SRH care services in Mysore, India and Mombasa, Kenya; 1) the extent to which payments are regressive, 2) the extent to which payments are catastrophic, and 3) explore coping mechanisms used to finance health spending.

## Materials and methods

### Participants and data collection

Data for this analysis were collected as a part of the baseline situational analysis for the “Diagonal Interventions to Fast Forward Enhanced Reproductive Health” (DIFFER) project. The DIFFER interventions aim to improve access to SRH and HIV services among women in general and Female Sex Workers (FSWs), and are being tested in four countries; Mombasa (Kenya), Durban (South Africa), Tete (Mozambique), and Mysore (India). The situational analysis aimed to describe baseline service provision and access, including the demographic and socio-economic characteristics of SRH service users at sentinel facilities. Information on service provision was collected through a detailed service audit not reported in this paper. Service access and the characteristics of service users were measured using a quantitative patient exit interview. There are a total of 13 sentinel facilities for which data were collected; three in India, four in Kenya, five in Mozambique and one in South Africa. These facilities offer a range of SRH services including care for sexually transmitted infections (STIs), family planning, HIV testing and counseling, HIV care and ART, cervical cancer screening, services for victims of gender-based violence, and termination of pregnancy and/or post abortion care.

Respondents eligible to participate in the patient exit interview for the situation analysis were women older than 18 years, who had completed a visit at one of the sentinel facilities, all urban centers, and were willing to sign an informed consent form. Respondents were recruited and interviewed immediately following their consultations for SRH services. The sample size of female SRH users was powered to enable the study to detect an increase in the proportion of satisfied health service users from 60 to 80 %, at the 95 % confidence level. The *sampsi* command in Stata/IC 11.0 for a two-sample comparison of proportions was used. The estimation was based on the method of Fleiss, Levin and Paik [22] to estimate the sample size to achieve a given power of a two-sided test for the difference in two proportions. Across the four study sites, a total of 614 female SRH users were interviewed (India: 150, Kenya: 100, Mozambique: 99, South Africa: 265). However, data on household income and coping mechanisms were not collected within the patient exit interviews in South Africa and Mozambique. As such, only the survey data collected in India and Kenya (*n* = 250 SRH users) are included in this study and described further below.

In India, data were collected from women attending three facilities in Mysore city. These facilities included two government hospitals (Cheluvumba and SMT Hospitals) and one private facility (Asha Kirana Hospital). 50 participants were interviewed at each facility for a total of 150 participants. Study participants were recruited using a consecutive sampling technique. In other words, all individuals who meet the selection criteria were approached to participate in the study. While the government facilities offered a range of different SRH services, Asha Kirana’s SRH services were limited to HIV care and treatment services. Accordingly, participants from the government facilities were chosen from the full range of different SRH services provided within the government facilities, but at Asha Kirana participants were limited to HIV positive women seeking HIV care and treatment.

In Kenya, a total of 100 participants were recruited from the family planning and STI clinical services at four representative urban public health facilities in city of Mombasa; two health centers (Kisauni and Chaani) and two district hospitals (Tudor and Likoni). With the help of health care providers, and with access to the health facility registers, the study team estimated the weekly number of clients using each SRH service (estimated at between 17 and 20 clients per service). Based on this estimate, 3 to 5 clients were interviewed per available service and up to 25 clients per facility in order to gather data on the full range of SRH services. Using an interval sampling method (or nth person selection technique) every 4th client was recruited as they exited each of the SRH services of interest.

The surveys collected data on the socio-economic characteristics of respondents including age, education level, occupation, relationship/s status, place of residence, average monthly household income, number of children, religion, the services requested and those provided to them, total expenditure, including both direct medical payments (i.e., payments for any services received at the facility, medicine, tests, consultations etc.) and direct non-medical payments (including the transportation costs), travel time, waiting time at the facility, sources of financing these expenditures, degree of satisfaction with services received, perceived unmet needs, and measures of empowerment and agency.

The questionnaire for the patient exit interview was designed to collect data through face-to-face interviews. The questionnaire was translated into local languages and then back translated into English to ensure accuracy. Respondents could choose to be interviewed in English or the local language. Interviews were conducted in December 2012 in India and between November 2012 and March 2013 in Kenya. Interviews were done by trained staff, with experience in research of this nature and who are used to asking sensitive questions. The study was reviewed and approved by the local Research Ethics Committee in each site as well as the ethics committee at Ghent University as the coordinating partner. Informed consent was given by all participants before they were enrolled in the study.

#### Data management and analysis

Data collected from the exit-interviews were entered into a Microsoft Access database, cleaned and extracted to Stata, Version 12, for analysis. Information on costs of seeking care, sources of financing (coping mechanisms), average monthly household income and demographic variables were extracted from the database for the analysis.

Average monthly household income was used as the measure of socioeconomic status. This included income generated by all members of household from different sources (including government grants, pension etc). Household was defined in this study as “all the people who live under one roof or who eat from a common pot”. Quintile ratios and a Kakwani index were used to measure the progressivity of the costs of seeking SRH services. To calculate quintile ratios, the proportion of health spending in the lowest and the highest income quintiles were compared and tested for significant differences in means using a *t*-test, using a 95 % confidence interval [12]. If individuals in the lowest income quintile bore more costs than those in the highest when seeking care, spending was defined as strongly regressive. If there was no significant difference in expenditure - if the lowest quintile did not spend significantly more or less - spending was defined as weakly regressive [12].

Spending was further analysed using the Kakwani index [23, 24]. This index is a commonly used measure of equity in health care financing/payments [6, 25–27], which compares the distribution of health care spending, plotted on the concentration curve, with the distribution of income or consumption expenditure (plotted on the Lorenz curve). The Kakwani index is defined as twice the area between the concentration curve and the Lorenz curve and is calculated as;

$$ \uppi \mathrm{k} = \mathrm{C}\ \hbox{-}\ \mathrm{G} $$

Where:

C = the health spending’ concentration index

G = the Gini coefficient of the household income.

The value of the Kakwani index (πk) ranges from -2 to 1. A negative index indicates regressive spending as the concentration curve lies inside the Lorenz curve, while a positive index indicates progressive spending as the concentration curve lies outside the Lorenz curve. When the index is zero, i.e. the concentration lies on the top of the Lorenz curve, the spending is proportional [24].

Catastrophic spending was calculated as the percentage of household income spent on SRH care seeking. There is no single accepted threshold for catastrophic health care payments. The rationale behind catastrophic spending is that households must reduce spending on their basic needs, and they might go into debt or sell productive assets, risking household livelihoods, over a period of time to cope with this degree of health care spending. While the choice of the threshold is effectively arbitrary, two commonly used thresholds are 10 % of total income [1, 28, 29] or 40 % of income after spending on food (referred to as capacity to pay) [2]. The former threshold (10 % of income) is employed for this analysis and we calculated the % of household income spent on the last reported SRH care event.

Further, the strategies that were adopted by the respondents in each site to cope with the costs of seeking care were analysed considering socio-economic status of the individuals.

## Results

### Socio-economic characteristics of the sample population

**Fig. 1:**
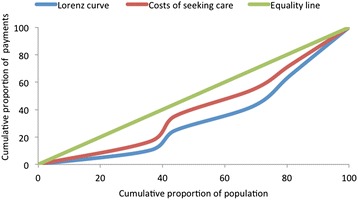
Concentration curves of costs of seeking care and Lorenz curve of household income, India

**Fig. 2:**
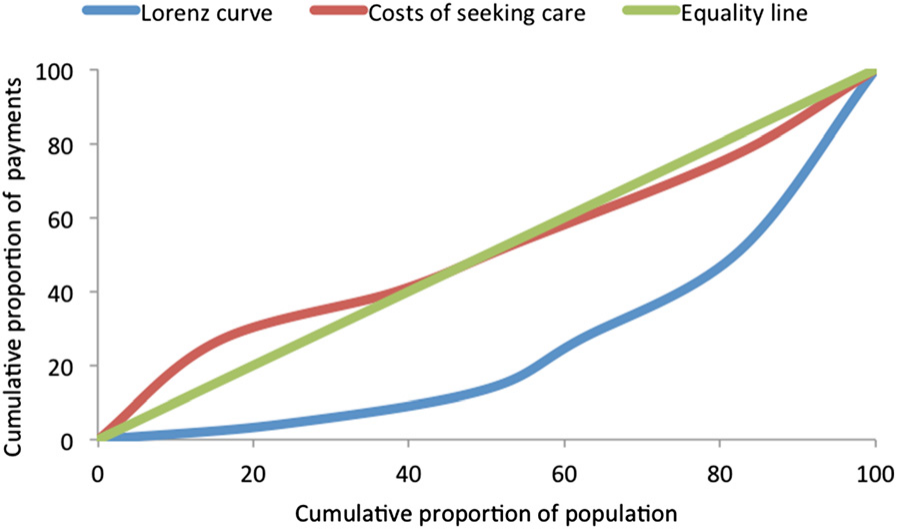
Concentration curves of costs of seeking care and Lorenz curve of household income, Kenya

Table 1 describes the socio-economic characteristics of female SRH users at the study site facilities. The mean age of respondents in India and Kenya were 27 and 30 years old, respectively. Nearly a third of respondents in India had never attended formal education (32.7 %), while this proportion was only 2 % in Kenya. Nearly all respondents in India were married and living with their partner (97.3 %), compared with 60 % of respondents in Kenya. Respondents had a median number of two children in both sites. Average household income in the least poor, compared with the poorest quintile, was ten times and 12 times higher in India and Kenya, respectively. Figures 1 and 2 illustrate the high income inequality between quintiles in both sites. In India, only 20 % of respondents had some form of employment, while in Kenya, this figure was 51 %.

**Table 1:** Socio-economic characteristics of the female SRH users in Mysore, India and Mombasa, Kenya

Description	India (*n* = 150)		Kenya (*n* = 100)	
	No	%	No	%
Mean (Median) age	27 (26)		30 (30)	
Age group				
18–20	28	19	4	4
21–25	47	31	23	23
26–30	29	19	29	29
31–35	19	13	22	22
>36	27	18	22	22
Education level				
Illiterate	49	33	2	2
Primary education	48	32	27	27
Secondary	43	29	52	52
University/college education	10	7	19	19
Current employment status				
Full/part time/self-employed	30	20	51	51
Unemployed^a^	120	80	49	49
Present relationship				
Married – living together	146	97	60	60
Married – living apart	3	2	8	8
Not married, living with partner	0	0	4	4
Single, no current partner	1	1	12	12
Separated/divorced	0	0	8	8
Other	0	0	7	7
Median Number of children	2		2	

^a^Including housewives (13 women in India and 10 in Kenya) and students/scholar (5 women in Kenya)

### Services received by female SRH care users

Table 2 presents the services received by participants during their visit. In India, the main services women received during their visit were STI diagnosis, treatment or follow-up (35 %) followed by general women’s health services (23 %) and HIV care (21 %). In Kenya, the main services received by women were family planning (26 %), STI diagnosis, treatment or follow-up (23 %) and HIV counseling and testing (21 %).

**Table 2:** Services received by the female SRH users

	India		Kenya	
Services received at the facility	No	%	No	%
PHC services	1	0	5	5
HIV counseling and testing (HCT)	26	12	24	22
Family planning	3	1	29	27
STI diagnosis/treatment/follow-up	73	35	26	24
Came for HIV care or ART follow up	44	21	22	20
SGBV counselling and related services	0	0	1	1
Pregnancy counseling, CTOP services and/or referral	5	2	3	3
General women’s health services (e.g. cervical cancer screening/follow-up)	48	23	0	0
Other	11	5	3	3

*SRH* Sexual and Reproductive Health, *PHC* Primary health care services; *CTOP* Choice on Termination of Pregnancy, *SGBV* Sexual and Gender Based Violence, *STI* Sexually Transmitted Infection, *ART* Antiretroviral Therapy

### Costs of seeking care

Table 3 presents the cost of seeking care for SRH services in India and Kenya. This is the cost of the care seeking event that took place immediately before the exit interview. In India, 18 women (12 %) reported payments for SRH services used, while in Kenya 41 women (41 %) reported these payments. In Kenya, respondents reported only payments to providers of SRH services (e.g., consultation, drugs etc.), while in India they only reported non-medical direct costs such as transportation costs and not costs paid for services. This is discussed later in the paper as a limitation of the analysis. The average cost of seeking care was approximately the same i.e. approximately 1USD in both sites. In the local currency this was Rs 57 in India and 82 Kshs in Kenya. On average, Indian respondents paid 1.3 % of their monthly household income for care, while this was 0.6 % for Kenyan respondents. Neither suggests that this single care seeking event was catastrophic or likely to be impoverishing.

**Table 3:** Cost of seeking care for the female SRH users^a^

	India (Rs) (*n* = 140)	Kenya (Kshs) (*n* = 41)
Monthly household income^b, c ^		
Mean (SD)	5167 (2674)	19582 (17609)
Median	5000	12000
Range	1000–20000	1500–80000
Seeking care costs		
Mean (SD)	57 (46)	82(104)
Median	40	30
Range	5–250	20–400
Mean spending in each income quintile		
Most poor	48.55	108.33
Very poor	53.30	55.56
Poor	56.86	53.33
Less poor	52.06	98.18
Least poor	78.50	94.29
Spending % of income		
Mean (SD)	1.3 % (1.1 %)	0.6 % (1.3 %)
Median	1 %	0.20 %
Range	0.1–6 %	0.03–8 %

^a^Spending for India is only included transportation costs and for Kenya is only included payments for SRH services

^b^Monthly household income is not adjusted for household size

^c^One US Dollar in 2012 was on average equal to 53.44 Indian rupees and 84.53 Kenyan Shilling

The progressivity of spending was then assessed using a Kakwani Index and a Quintile Ratio as described in the previous section. Figures 1 and 2 show the concentration curves for income and costs of care seeking distributions. These curves, and the Kakwani indices calculated, demonstrate that spending on sexual and reproductive health services was highly regressive in both sites (*k* = -0.13 for India and *k* = -0.49 for Kenya). As such, households in lower income quintiles spend a higher percentage of their income on care compared with those in higher income quintiles. There were also significant differences in the proportion of payments across the income quintiles. The proportion of income spent on seeking care was two times and ten times higher among the poorest households in India and Kenya respectively, compared with the least poor households. This difference was statistically significant (*p-value* = 0.001 and 0.006 for India and Kenya, respectively).

### Sources of finance (coping mechanisms)

In India, care seeking payments were most commonly financed with money given by partners or other household members (69 %). In Kenya, these payments were most commonly financed with money from own savings or regular income (44 %) followed by no coping strategy (26 %), and money given by partners or other household members (25 %). Details of sources of finance are described in Table 4. In both sites, there was a significant difference in financing method by employment status of the respondent, indicating unemployed respondents mainly received money from their partners or a household member, while employed respondents used their savings or salary to finance the costs of seeking care (*p* = 0.000 and *p* = 0.002, India and Kenya, respectively). There was no significant relationship between the coping strategy adopted and income level, age group or marital status in either sites. In Kenya, there was a significant difference (*p* = 0.012) in the coping strategy by education level (i.e. women with the secondary and higher education used mainly their savings or regular income, whereas less educated women received money from their partners or family members). However, differences by education level were not significant in India (*p* = 0.163).

**Table 4:** Sources of financing the costs of seeking care (coping mechanisms)

Coping strategy	India		Kenya	
	No	%	No	%
Own savings/regular income	32	21	44	44
Partner/household member gave the money	104	69	25	25
Own savings/regular income & Partner/household member gave the money	7	5	0	0
Family member not living in the same household gave me money	2	1	2	2
Loan from household member that must be repaid	4	3	1	1
Loan from someone not living in the household that must be repaid	0	0	1	1
No coping strategy used	0	0	26	26
Other	1	1	1	1
Total	150	100	100	100

## Discussion

This study aimed to assess inequity in spending on SRH services in India and Kenya. Specifically, this study assessed: 1) progressivity of payments, 2) the extent to which payments are catastrophic, and 3) the coping mechanisms adopted by the respondents to finance the payments. The findings showed that the spending on SRH services was highly regressive in both sites, with the lower income households spending a significantly higher percentage of their income on seeking care compared to households with a higher income. In India, the poorest households spent two times and in Kenya ten times more on seeking care than the least poor households. However, expenditure on this single care-seeking event was not catastrophic or likely to be impoverishing. The findings also indicated that the common coping mechanisms adopted by respondents in India and Kenya were “received from partner or household members” and “own saving or regular income”, respectively.

The finding that spending was highly regressive is in line with findings from national studies describing the regressive nature of total health care financing/payments in these two countries [30, 31]. India and Kenya have different health systems structures in that India has a decentralised public health system, while Kenya’s system is highly centralized [30, 31]. However, both settings are pluralistic, with a significant proportion of services offered either by private providers or by public providers charging users fees at the point of use. In both settings, out of pocket spending constitutes a high proportion of total health expenditure; approximately 60 % in India and 50 % in Kenya [32, 33]. The government contribution to total health spending is similarly low in both contexts; approximately 32 % in India and 42 % in Kenya [34]. Considering the high proportion of OOPE in both countries, the findings of these analyses are perhaps unsurprising.

Although the findings of this study don’t suggest that the cost of a single care seeking event was catastrophic or likely to be impoverishing, this may not be the case if multiple visits are required. For some services, patients or service users are required to have repeated visits to the facilities (for example, repeated visits for family planning, or HIV care and treatment services). Relatively small repeated payments can result in financial catastrophe forcing households into poverty. Evidence from previous studies has shown that patients may delay seeking care or fail to seek care because of the high cost of services or high opportunity costs of care seeking [29, 35, 36]. Moreover, other studies in India and Kenya have shown the negative impact of user fees on utilization of healthcare services and incidence of catastrophic spending, including SRH [30, 33, 37]. The high rate of transport spending, in particular in the Indian site, suggests that, while user fee exemption may offer some protection from catastrophic health expenditure, a significant and regressive financial barrier to service access remains. Moreover, the opportunity cost of travelling and time spent at the facility can act as a barrier to accessing services. The findings show that in India, median time taken to reach the facility and time from arriving until completing a visit at the facility was 60 and 120 min, respectively. In Kenya, respondents took an median of 20 min to reach the facility and 60 min to complete their visit.

The literature suggests that households use various strategies to cope with health shocks, which can differ in different contexts [7–9, 38, 39]. Generally, coping strategies are categorised into two broad categories; detrimental and non-detrimental. Detrimental or harmful strategies include strategies such as borrowing money/taking out loans or selling productive assets. Non-detrimental strategies commonly include the use of income and savings, labour substitution or social networks [7–9, 38, 39]. Detrimental coping strategies can have a dramatic impact on future earnings and may leave households at higher risk of increased economic vulnerability and poverty in the long run [7–9, 38]. Non-detrimental strategies, such as income and savings, are generally used when health spending is small or moderate. Although these funding sources are commonly referred to as non-detrimental in the literature, it should be noted that this spending can have a temporary, negative impact on household finances and consumption [7]. In poorer households this negative effect may be significant and this warrants further exploration in future empirical work.

The findings of this study indicated that the most common coping mechanisms adopted by respondents in India was “receiving money from partner or household members” and in Kenya was “using own saving or regular income”. This finding can be explained by the unemployment rate of the respondents in both sites. If the money from partners or household members does not need to be repaid, then these would generally be considered non-detrimental strategies. Using detrimental coping strategies was rare among respondents in both sites, where very few borrowed money and no one sold productive assets.

Furthermore, previous studies have shown that lack of financial control can limit women’s access to SRH services [40, 41]. Based on the results of this study, a significant proportion of respondents (particularly in India with more than 70 %) were unemployed. This means that they depend on their spouses or other household members to pay for health expenditure. This may discourage them from seeking SRH-related care on time or at all. Further, the large proportion of service users relying on borrowing to finance care seeking suggests that care access would be more difficult for those with weak social ties, small social networks or weak bargaining positions within the family - although this requires further investigation. It is well documented that in LMICs countries, social networks are one of the most important resources mobilized by households to obtain money to seek treatment [9, 29, 42, 43], although some evidence suggests that the poorest have the weakest social resources [29, 42, 43].

The results from this study should be interpreted in light of a number of limitations. Firstly, this study only included women who sought out and accessed health services. As such, the results might not be generalizable to the wider population of the countries under study. In addition, although the facilities from which respondents were recruited, are considered to be representative of urban facilities in both countries, the composition of patients visiting the facilities may differ from other urban facilities. Secondly, due to the small sample size, it was not possible to conduct robust sub-sample analyses and the results of the analyses presented (i.e. association of the coping strategies and socio-economic status), should be interpreted with caution. Thirdly, monthly household income is not adjusted for household size, as we didn’t have an appropriate measure of household size. Fourthly, expenditure for Kenya included only payments to the providers of SRH services and for India, it is only included non-medical direct costs such as transportation costs. In India, 18 respondents (12 %) stated that they were asked to pay for services however, they did not report the amount paid. Although the effect on the magnitude of inequality or degree of regressiveness of spending (i.e. the main focus of this study) is not clear, if full costs had been measured, total expenditure had been higher, so we had achieved a better picture of the likelihood of catastrophic payments. Finally, this study did not measure the opportunity costs of seeking care (i.e. lost earning opportunities due to time spent seeking care). Opportunity costs are widely considered to be a sizeable component of the overall costs of health care in contexts such as these. However, due to difficulties quantifying these costs, in common with many other studies, they were not included in this study. As such, our estimates of spending are highly conservative in nature.

## Conclusion

These findings demonstrate that spending on SRH services are highly regressive in India and Kenya. Highly regressive spending on SRH services highlights a heavier burden borne by the most poor when seeking care in these resource-constrained settings. The findings can provide useful information for policy makers and programme managers in designing appropriate policies to improve access to care, reduce socio-economic inequality, and promote progress towards the MDGs in these settings.

## Abbreviations

LMICs: Low and middle income countries
SRH: Sexual and reproductive health
DIFFER: Diagonal Interventions to Fast Forward Enhanced Reproductive Health
OOPE: Out-of-pocket expenditures
WHO: World Health Organization
MDG: Millennium Development Goals
